# Evaluation of Planetary Health and Sustainable Healthcare Online Course for Undergraduate Medical Students: A Quasi-experimental Study

**DOI:** 10.1007/s40670-025-02462-0

**Published:** 2025-07-14

**Authors:** Hanaa Saeed Elhoshy, Soha Rashed Aref, Mennatallah Hassan Rizk

**Affiliations:** 1 Department of Medical Education, Faculty of Medicine, Alexandria University, Alexandria, Egypt; 2 Department of Community Medicine, Faculty of Medicine, Alexandria University, Alexandria, Egypt

**Keywords:** Sustainable Healthcare, Planetary health, Climate change, Undergraduate medical curriculum, Online learning, Community of inquiry

## Abstract

**Introduction:**

Introducing medical students to sustainable healthcare and planetary health concepts is essential to preparing future physicians for the growing environmental challenges that impact human health. This study evaluates the effectiveness of an online elective course on sustainable healthcare and planetary medicine, designed to align with established sustainable healthcare priority learning outcomes.

**Methods:**

This study used a one-group pretest–posttest design to evaluate an online course on planetary health and sustainable healthcare for third-year medical students at Alexandria University. The community of inquiry (CoI) framework guided course development. A non-probability sample of 206 students was recruited. Due to the short-term nature of this study, evaluation focused on the first two levels of Kirkpatrick’s model: level 1 (reaction) used a satisfaction survey to measure engagement and effectiveness, and level 2 (learning) used a 20-item pretest–posttest exam to assess knowledge acquisition. The pretest also captured students’ perceptions of their prior knowledge and attitudes toward incorporating planetary health into medical education.

**Results:**

Before the course, only 29.1% of students perceived they had sufficient knowledge of sustainability in healthcare, while 93.2% expressed a need for more education in this area. At the end of the course, all students passed the 20-item posttest, with a statistically significant improvement in posttest scores compared to pretest scores (*p* < 0.05). Students reported the highest satisfaction with clarity of grading criteria, achievement of learning outcomes, instructor communication, response time for feedback, and overall course quality.

**Discussion:**

The findings align with outcomes reported in comparable courses, demonstrating consistent improvements in student knowledge and engagement in planetary health and sustainable healthcare education.

**Conclusion:**

The online elective course on planetary health and sustainable healthcare significantly enhanced students’ knowledge and was well received, as evidenced by high satisfaction ratings. However, areas for enhancement include improving multimedia content, expanding course topics, and providing additional supplemental materials.

**Trial registration:**

Not applicable.

## Introduction

As the world grapples with climate change and environmental sustainability, a broader concept of health has emerged—planetary health—which emphasizes the interdependence between human health and the health of the planet’s ecosystems [1, 2]. This transdisciplinary field explores how environmental degradation impacts public health and seeks solutions that integrate sustainability into healthcare systems [4, 5].

Ironically, the healthcare sector itself is a major contributor to environmental harm, accounting for approximately 4.4% of global greenhouse gas emissions [6, 7]. Sustainable healthcare, defined as minimizing environmental impact while ensuring equitable and effective health services, offers a strategic approach to mitigating this issue [8]. It aims to balance high-quality patient care with resource efficiency and environmental stewardship, ensuring long-term sustainability in healthcare delivery [9].

On the medical education side, there are calls for preparing undergraduate medical students to adapt to the expected changes in human health and wellbeing [10]. As a result of this recognition, there is a rising effort to incorporate planetary health and sustainable healthcare education into undergraduate education in medical schools. This educational transition is critical in educating future healthcare professionals to understand and address the interdependence of health and ecosystems, as well as to respond effectively to the health problems of climate change [10]. This shall also equip healthcare professionals with the knowledge and skills to address environmental health issues and foster a culture of sustainability within the healthcare sector and encourage the adoption of planet-friendly practices [10]. Recently, the General Medical Council (GMC) of the UK requires the integration of education for sustainable healthcare (ESH) into medical curricula, stressing the importance of equipping students with the knowledge and skills to incorporate sustainable practices into their medical careers [11].

Despite the pressing need, planetary health education remains largely missing from medical curricula. A survey of over 100 countries revealed that only 15% of medical schools address climate change and air pollution in their curricula [12]. The Planetary Health Report Card (PHRC) is a 2-year student-led, metric-based evaluation tool designed to assess the extent to which medical schools engage with and integrate planetary health and sustainability into their operations and curricula. It systematically evaluates institutions across five core domains: curriculum, research, community outreach and advocacy, support for student-led initiatives, and campus sustainability. The PHRC utilizes a multilevel point system in which each metric is assigned a maximum value of 2 to 3 points. These scores are then converted into percentage values, translated into letter grades using a standardized grading scale, and reflected levels of integration: A (80–100%, excellent), B (60–79%, good), C (40–59%, insufficient), D (20–39%, poor), and F (0–19%, nearly absent/absent). In year 1 (2019–2020), the PHRC evaluated 13 medical schools in North America, yielding an overall average score of 63%, corresponding to a B grade. However, performance within specific domains revealed notable deficiencies, with the curriculum receiving a score of 52%, corresponding to a C grade. In year 2 (2020–2021), the assessment expanded to include 62 medical schools across five countries [13]. Strikingly, 98.4% of these institutions received an overall grade of C or lower, highlighting a pervasive lack of integration of planetary health and sustainability across global medical education systems [13]. In the UK, although sustainable healthcare is a regulatory requirement, 33.3% of medical schools participating in the Planetary Health Report Card (PHRC 2022–2023) did not integrate it into their core curriculum [14].

Several barriers hinder the integration of planetary health into medical education, including curriculum overload, lack of faculty expertise, and limited instructional resources [6]. Traditional medical curricula, often siloed and dominated by the biomedical model, do not adequately address environmental and social determinants of health, making interdisciplinary sustainability education challenging [15]. Additionally, ethical and equity considerations add complexity to integrating planetary health into healthcare training [3]. Overcoming these barriers requires faculty training, curriculum reform, and interdisciplinary approaches to embed sustainability in medical education effectively [6, 15, 16].

Despite these challenges, innovative teaching strategies and digital learning platforms offer promising solutions [17]. Case-based learning (CBL) has proven effective in illustrating the impact of environmental factors on health, fostering critical thinking and problem-solving skills [18]. This approach enhances critical thinking, encourages problem-solving, and helps future healthcare professionals understand the urgency of integrating sustainability into medical practice [18]. Additionally, online learning environments provide a flexible and scalable method for incorporating planetary health education into medical programs [14]. Resources such as the Planetary Health Alliance’s Education Framework, which offers open-access multimedia and specialized databases, can help educators enrich their curricula and enhance student engagement [19].

While global and regional literature—particularly within the African context—has clearly identified significant gaps and issued urgent calls for the integration of sustainability and planetary health into medical education, there is a notable absence of published studies that examine these issues within the Egyptian undergraduate medical curriculum [8, 20–22]. In addition, the urgent need to build workforce capacity to address the health impacts of climate change was underscored at **COP27**, the 27th United Nations Climate Change Conference, an international event hosted by **Egypt in Sharm El-Sheikh in 2022**. For the first time, health was prominently featured on the agenda, with the World Health Organization supporting a dedicated Health Pavilion. Key outcomes included strong calls to integrate climate change into health professional education, strengthen climate-resilient health systems, and mobilize healthcare workers as agents of sustainable and preventive care [24, 25]. These global recommendations are directly relevant to the Egyptian context, where formal education on planetary health and sustainability remains limited in undergraduate medical training. Specifically, no research was found that analyzes existing curricular gaps or provides recommendations tailored to the Egyptian context. Given the need to bridge the knowledge gap in planetary health education specially with the Egyptian context, this study evaluates the effectiveness of an online elective course on planetary health and sustainable healthcare offered to third-year medical students at Alexandria University, Egypt. To achieve this aim, specific objectives have been outlined: first, to assess students’ baseline knowledge and perceptions of planetary health and sustainable healthcare before the course; second, to evaluate the course’s effectiveness in enhancing students’ knowledge acquisition by measuring pretest and posttest scores; third, to gauge students’ satisfaction levels regarding the course content, teaching methods, and overall learning experience; and fourth, to identify areas for improvement in the course structure and content based on student feedback. The research hypotheses postulate that participation in the online elective course will result in a statistically significant improvement in students’ knowledge, as indicated by pretest and posttest scores. Additionally, it is hypothesized that students will report high levels of satisfaction with the course structure, content, and delivery. Finally, it is expected that students will enter the course with varying levels of perception regarding the relevance of planetary health and sustainable healthcare to medical education, influenced by their prior awareness of environmental and health-related issues.

## Methods

### Study Design, Setting, and Participants

This study employed a quasi-experimental one-group pretest–posttest design to evaluate an online elective course on planetary health and sustainable healthcare within the medical school curriculum. The course aimed to equip third-year medical students with fundamental knowledge on key environmental challenges in Egypt, their impact on human health, and sustainable healthcare solutions. The learning objectives focused on four main topics: planetary health, sustainable healthcare, water scarcity in Egypt, and climate change and its health impacts. An online teaching and learning strategy was implemented using the community of inquiry (CoI) framework, which is a widely adopted model for designing and evaluating effective online and blended learning environments in medical education [26]. The CoI framework emphasizes the interplay of three essential elements: teaching presence, which involves the design, organization, and facilitation of learning activities; social presence, which enables learners to communicate openly and develop a sense of trust and community; and cognitive presence, which refers to learners’ ability to engage in critical thinking and construct meaning through reflection and dialogue. By integrating these elements, the CoI framework supports the development of interactive, learner-centered virtual courses that foster engagement, collaboration, and deeper understanding, which are key components in the context of medical education [27]. The Moodle platform was used for structured learning, including recorded asynchronous lectures, synchronous Zoom meetings, and interactive discussions via WhatsApp groups. For the teacher’s presence, lectures were delivered asynchronously via recorded videos, supplemented by synchronous Zoom meetings for real-time interaction. The social presence was fulfilled through a dedicated WhatsApp group, and Zoom meetings facilitated group discussions and group assignments, fostering a sense of community among students and instructors. Cognitive presence was encouraged through group activities like designing infographics and developing concept maps on health issues related to climate change, promoting critical thinking and deeper understanding. The three presences overlapped through course daily tasks, either synchronous on Zoom or asynchronous on Moodle with daily WhatsApp reminders. Moreover, the course incorporated group research projects, where students explored novel sustainability topics and presented findings through infographics, receiving structured feedback from instructors.

The course lasted for 12 days during the 2022–2023 academic year, with a total workload of 15–20 h, including self-directed activities and assignments. The instructional team included a full professor of community medicine, one assistant professor of medical education, and two teaching assistants from the Department of Medical Education, ensuring both subject matter expertise and pedagogical support throughout the course. The total instructional content comprised two lectures and a two-phase project assignment. The course consisted of a blended learning format, with one interactive Zoom session serving as the primary synchronous component, and one pre-recorded lecture providing asynchronous content. The core of the course is centered on an independent, student-led research project supported asynchronously through structured feedback on Moodle and real-time communication via a designated WhatsApp group. Knowledge assessment was conducted using identical pretest and posttest instruments, each comprising multiple-choice questions designed to evaluate the lower levels of Bloom’s Taxonomy, specifically recall and comprehension. Higher-order cognitive skills, including applying, analyzing, and creating, were assessed through the course project. Detailed description and organization of course activities are presented in Table 1.

**Table 1:** Detailed description and organization of course activities

Activities	Description
Day 1: course joining and orientation Moodle login WhatsApp group joining Pretest taking Orientation video watching	Initial on boarding tasks to ensure active participation, easy communication, and survey of basic knowledge and practices using the pretest using A 20 MCQs pretest and a questionnaire
Day 2: climate change and planetary health Synchronous Zoom interactive lecture and course project introduction	Synchronous lecture covering basic definitions of climate change, its effects of human health, different body organs, effects on ecosystem, definition of planetary health and magnitude of current problem and sustainable healthcare definition as a suggested healthcare strategy to improve planetary health. Additional readings and videos were available on Moodle Comprehensive instructions for the course project, outlining its three main phases: researching emerging topics in planetary health and sustainable healthcare, developing concept maps and infographics, and adhering structured submission timelines with both formative and summative deadlines were included at the end of the lecture. The concept maps and infographs were illustrated with examples, guidance on using digital platforms for creation was offered, and the use of credible academic sources for research was emphasized. It also confirmed the WhatsApp group as the primary channel for group assignments, topic distribution, and ongoing support. Instructional video resources were made available via Moodle, including YouTube tutorials on concept mapping, guidance on creating infographics using the Piktochart platform, and a recorded orientation session to support students’ independent project work
Days 3 to 6 Course project active phase	During the active phase, students collaborated in assigned groups with ongoing instructor support, engaging in flexible, self-paced project work designed to deepen learning and ensure timely feedback. The selected topics were novel and extended beyond the scope of the lecture content, allowing students to independently explore emerging themes in planetary health and sustainable healthcare through guided research. Students were divided into small groups of no more than 10 students each. These groups were distributed evenly across four course instructors, who provided project supervision and feedback. Project topics included: impact of water scarcity on healthcare access, eco-friendly medical waste, climate change and infectious diseases, effect of air pollution on central nervous system, innovative technologies for sustainability in healthcare, the role of healthcare in carbon emission, training for promoting sustainable healthcare, impact of climate change on public health, telemedicine and environmental sustainability, effect of climate change on elderly, the role of physicians in sustainability, reducing medical waste, water conservation in healthcare systems, climate change and malnutrition, climate change and cardiovascular health outcomes, climate change and respiratory health outcomes, renewable energy and healthcare, climate change and child health impact, climate change and women health, climate change and mental health, and health recommendations of 28th Conference of the Parties (COP 28) to the United Nations Framework Convention on Climate Change (UNFCCC)
Day 7 Lecture 2 video and soft deadline for formative feedback	Asynchronous recorded lecture via Moodle covering basic definitions of water scarcity, magnitude, impact on health and solutions of the problem within the Egyptian context The soft deadline for formative feedback was implemented through a Moodle assignment submission, allowing students to upload their draft project materials for instructor review and receive constructive feedback prior to final submission
Course project revision phase	Students received detailed formative feedback on their initial project submissions. Formative feedback was provided through the Moodle platform using assignment comments, as well as privately to each group via WhatsApp. This feedback addressed three key areas: the depth and clarity of topic coverage, the structure and accuracy of concept maps, and the effectiveness of infographic design. The goal was to guide students in refining their work by highlighting strengths, identifying gaps, and suggesting improvements. Students were then given the opportunity to revise their projects based on this feedback, reinforcing a process-oriented approach to learning and encouraging critical reflection and iterative development
Day 11: course final exam (posttest)	The final course exam consisted of 20 multiple-choice questions (MCQs) targeting lower levels of Bloom’s Taxonomy, designed to assess recall and understanding foundational knowledge across all course topics and objectives; higher-order cognitive skills were evaluated through the course project
Day 12: final deadline for assignment and course evaluation form	The summative infographic submission was evaluated using a 5-point rubric encompassing 4 key criteria: the accuracy and depth of content, the clarity and organization of the infographic’s conceptual structure, the visual design and communicative effectiveness of the infographic, and the appropriate use and citation of academic sources. While the research process and concept maps were essential preparatory components that supported the development of the final product, they were not included in the summative assessment. Additionally, students completed a course evaluation form, which served to assess perceived learning outcomes and gather feedback for ongoing course refinement and improvement

### Sampling

A non-probability convenience sampling method was used to recruit third-year medical students from the Faculty of Medicine, Alexandria University. Enrollment was voluntary and open to all students in their third year, allowing participants to self-select on the course. A total of 206 students participated, representing 7.99% of the total third-year student population (*N* = 2587). Given the exploratory nature of the study and limited prior research on the topic, a sample size calculation was conducted using G*Power version 3.1.9.7 (University of Düsseldorf) to determine the minimum number of participants needed to detect a statistically meaningful effect. Assuming a medium effect size (Cohen’s *d* = 0.5), an alpha level of 0.05, and statistical power of 0.80, the required sample size was estimated to be 45 participants. Despite employing a non-probabilistic sampling method, the final sample included 206 participants, substantially exceeding the calculated minimum. Furthermore, the observed effect size was very large (Cohen’s *d* = 2.19), calculated using Psychometrica’s effect size calculator which confirmed that the study was adequately powered to detect within-group changes [28].

### Data Collection Methods and Tools

The course’s effectiveness was evaluated using Kirkpatrick’s four-level model, which is a comprehensive framework for evaluating the effectiveness of educational and training interventions [29]. The first level, reaction, assesses learners’ immediate responses to the educational experience, including their satisfaction with the course content, delivery, and overall engagement. This level is important for understanding how well the training was received and whether it met participants’ expectations. The second level, learning, evaluates the extent to which participants have acquired the intended knowledge, skills, attitudes, or confidence as a result of the intervention. This is typically measured through assessments such as pretests and posttests or other performance indicators. The third level, behavior, examines whether the acquired knowledge or skills are being applied in real-world settings, such as clinical practice or academic environments, indicating the transfer of learning beyond the classroom. Finally, the fourth level, results, focuses on the broader impact of the training on organizational or systemic outcomes, such as improved patient care, policy change, or institutional advancement. While Kirkpatrick’s model includes four levels of evaluation, this study focuses on the first two levels, reaction and learning, as they are most appropriate for evaluating short-term educational outcomes within the scope of a single instructional intervention. For level 1, a self-administered online questionnaire was utilized to assess students’ perceptions of course quality and effectiveness based on the quality standards of online courses by Quality Matters (QM) [30]. A panel of six experts in medical education and public health conducted content validation, ensuring clarity, relevance, and alignment with course objectives. Feedback led to minor refinement to improve phrasing and alignment with planetary health concepts. The final questionnaire comprised 14 items rated on a 5-point Likert scale (1 = strongly disagree to 5 = strongly agree) and addressed areas such as perceived knowledge improvement, attitudes toward sustainability, instructor support and responsiveness, workload and participation, and overall course evaluation. For descriptive purposes, the mean satisfaction scores were categorized after analysis as follows: low satisfaction (1.00– < 3.00), indicating students were dissatisfied; intermediate satisfaction (3.00– < 4.00), suggesting students found the aspect acceptable but with room for improvement; and high satisfaction (4.00–5.00), where students were highly satisfied. To gather qualitative feedback on course strengths and improvement areas, two open-ended questions were included. The questionnaire demonstrated excellent reliability with Cronbach’s alpha of 0.97, ensuring consistency in responses.

For level 2, a 20-item multiple-choice exam assessed students’ knowledge acquisition before and after the course, where each correct answer earned one point for a maximum score of 20. The pre- and posttest had identical questions targeting lower levels of Bloom’s Taxonomy covering all course topics: definitions and causes of climate change, health effects of environmental changes, and sustainability practices in healthcare. Additionally, the pretest included background data (gender, nationality, prior exposure to planetary health concepts) and four Likert-scale questions that assessed students’ attitudes toward integrating planetary health into the medical curriculum and their perceived preparedness to tackle climate-related health challenges. Students’ pre-course sustainability practices were assessed using a 3-point Likert scale measuring the frequency of behaviors such as walking instead of using gas-powered vehicles, separating recyclable and non-recyclable waste, and seeking recycled products while shopping. The exam exhibited good reliability with Cronbach’s alpha of 0.78, indicating it was a consistent measure of knowledge gain.

### Data Analysis

Quantitative data from pretest–posttest scores and satisfaction surveys were analyzed using IBM SPSS (version 22.0, Armonk, NY: IBM Corp.). Descriptive statistics, including percentages, means, standard deviations, and 95% confidence intervals, were employed to summarize the findings. A paired sample *t*-test was utilized to determine whether posttest scores were significantly higher than pretest scores, with a *p*-value of less than 0.05 considered statistically significant. Subsequent categorization knowledge scores were categorized after paired as follows: low knowledge (0–10, ≤ 50% correct), moderate knowledge (11–15, 51–75% correct), and high knowledge (16–20, > 75% correct) was performed post hoc and for descriptive purposes to support interpretation of group trends. Additionally, qualitative responses from open-ended survey questions were analyzed with QDA Miner Lite V 3.0.6 (Provalis Research) to identify themes and subthemes related to students’ feedback.

## Results

A total of 206 third-year medical students participated in the Sustainable Healthcare course. Egyptian students comprised 59.7%, while 39.8% were Arab non-Egyptians (Kuwaiti, Sudanese, Saudi, Jordanian, Palestinian, Yemeni, Iraqi, and Syrian), and 0.5% were Canadian.

Before taking the course, students had obtained information about climate change from various sources. The most common sources were social media (86.9%) and internet searches (81.1%), followed by television (65%), courses (61.1%), the World Health Organization (WHO) (55.8%), and the Ministry of Health (MOH) (55.3%). However, only 29.1% of students perceived that they had sufficient knowledge about sustainability in healthcare, while 93.2% expressed a need for more education on this topic.

### Students’ Perceptions of Their Role in Addressing Climate Change

Regarding medical students’ role in addressing the health impacts of climate change, 84.5% believed they could take effective actions toward sustainability in healthcare, and 92.7% felt they must be well-prepared for their future roles as physicians. Additionally, 87.3% agreed that they could actively contribute to mitigating climate change, and an equivalent percentage believed that medical schools should prepare them to deal with climate-related health issues.

### Students’ Pre-course Attitudes Toward Integrating Planetary Health into the Medical Curriculum

As shown in Table 2, students rated the integration of planetary health elements into the medical curriculum as highly important. The highest-rated areas were climate change–related health impacts, clinical knowledge and skills, local geographic and climatic information, and emergency care needs related to climate change.

**Table 2:** Students’ pre-course attitudes toward integration of planetary health elements into medical curriculum

Integration of planetary health elements into medical curriculum		Count	%	*M* ± SD	Mean importance category
**Health impacts of climate change should be integrated into medical education curriculum**	Extremely important	118	57.3%	3.38 ± 0.852	High importance
	Moderately important	60	29.1%		
	Slightly important	17	8.3%		
	Not at all important	11	5.3%		
**Clinical knowledge and skills on climate change should be increased**	Extremely important	114	55.3%	3.40 ± 0.789	High importance
	Moderately important	69	33.5%		
	Slightly important	15	7.3%		
	Not at all important	8	3.9%		
**Local geographic and climatic information should be taught**	Extremely important	89	43.3%	3.20 ± 0.858	High importance
	Moderately important	80	38.8%		
	Slightly important	26	12.6%		
	Not at all important	11	5.3%		
**Emergency care needs related to climate change to be expanded**	Extremely important	123	59.8%	3.33 ± 0.962	High importance
	Moderately important	45	21.8%		
	Slightly important	21	10.2%		
	Not at all important	17	8.2%		

*M*, mean; *SD*, standard deviation; mean importance categories:low (1–2), intermediate (>2–3), and high (>3)

Students exhibited variable sustainability practices, as shown in Table 3. The most frequently reported practice was walking instead of using a gas-powered vehicle, categorized as moderate frequency. However, garbage separation and purchasing recycled products were less frequent practices among students.

**Table 3:** Students’ pre-course sustainability practices frequency

Sustainability practices		Count	%	*M* ± SD	Mean frequency category
**Walking instead of riding a vehicle that runs on gas**	**Always**	46	22.3%	2.14 ± 0.534	Intermediate frequency
	**Sometimes**	143	69.4%		
	**Never**	17	8.3%		
**Separating garbage to recyclable and non-recyclable**	**Always**	20	9.7%	1.77 ± 0.608	Low frequency
	**Sometimes**	119	57.8%		
	**Never**	67	32.5%		
**Looking for recycled products during shopping**	**Always**	18	8.7%	1.71 ± 0.617	Low frequency
	**Sometimes**	111	53.9%		
	**Never**	77	37.4%		

*M*, mean; *SD*, standard deviation; mean frequency categories: (low 1–2, intermediate >2–3, and high >3)

The paired sample *t*-test results, shown in Table 4, indicated that students’ posttest scores (mean = 16.69, SD = 1.51) were significantly higher than their pretest scores (mean = 12.41, SD = 2.31), with *p* < 0.05, confirming a statistically significant improvement in knowledge. This suggests that the course effectively enhanced students’ understanding of planetary health and sustainability in healthcare.

**Table 4:** Comparison of pretest and posttest scores

	*M* ± SD	Knowledge category	*t*	sig
Pretest scores	12.41 ± 2.31	Moderate	24.220	< 0.001*
Posttest scores	16.69 ± 1.51	High		

*M*, mean; *SD*, standard deviation; *t*,* t*−statistic; *p< 0.05 indicates statistical significance; knowledge scores: low (0–10, ≤50% correct), moderate (11–15, 51–75% correct), high (16–20, >75% correct)

### Students’ Satisfaction with the Course

All items in Table 5 received intermediate to high satisfaction ratings (mean > 4.00), indicating a strongly positive perception of the course structure, content, and instructor engagement. Instructor support (4.1 ± 1.29) and response time (4.1 ± 1.26) received high satisfaction, suggesting that students found instructors approachable, helpful, and responsive to their questions and concerns. Additionally, grading criteria clarity (4.00 ± 1.33) and learning activity relevance (4.0 ± 1.29) were rated highly, indicating that students found the course well-structured and aligned with learning objectives. Ease of online communication with peers was highly satisfying (4.1 ± 1.25) which suggests that the course effectively facilitated collaborative learning and interaction despite being delivered online. The highest-rated item was the overall course rating (4.4 ± 0.70), reflecting strong overall satisfaction and perceived value of the course.

**Table 5:** Students’ satisfaction levels toward course elements

Items	Strongly disagreed *n* (%)	Disagreed *n* (%)	Neutral *n* (%)	Agreed *n* (%)	Strongly agreed) *n* (%)	*M* ± SD	Mean satisfaction category
This e-learning sustainable health care course has increased knowledge of planetary medicine	23 (11.2%)	6 (2.9%)	16 (7.8%)	75 (36.4%)	86 (41.7%)	3.9 ± 1.28	Intermediate satisfaction
This e-learning sustainable health care course has helped to improve the skills required to advocate for planetary health	19 (9.2%)	10 (4.9%)	30 (14.6%)	70 (34.0%)	77 (37.4%)	3.8 ± 1.25	Intermediate satisfaction
This e-learning sustainable health care course had a positive impact on my attitude to the importance of sustainable	24 (11.7%)	8 (3.9%)	14 (6.8%)	67 (32.5%)	93 (45.1%)	3.9 ± 1.30	Intermediate satisfaction
The goals of this course were clearly stated at the beginning of the course	20 (9.7%)	5 (2.4%)	22 (10.7%)	70 (34.0%)	89 (43.2%)	3.9 ± 1.25	Intermediate satisfaction
My expectations for this course were met	17 (8.3%)	13 (6.3%)	29 (14.1%)	75 (36.4%)	72 (35.0%)	3.8 ± 1.23	Intermediate satisfaction
The grading criteria were clearly communicated at the beginning of the course	23 (11.2%)	8 (3.9%)	16 (7.8%)	55 (26.7%)	104 (50.5%)	4.0 ± 1.33	High satisfaction
The workload demands for this course were realistic for an online course	19 (9.2%)	10 (4.9%)	26 (12.6%)	50 (24.3%)	101 (49.0%)	3.9 ± 1.29	Intermediate satisfaction
The content of the videos was relevant to the learning outcomes of the course	24 (11.7%)	9 (4.4%)	10 (4.9%)	67 (32.5%)	96 (46.6%)	3.9 ± 1.30	Intermediate satisfaction
The learning activities were relevant to the goals of the course	22 (10.7%)	7 (3.4%)	13 (6.3%)	63 (30.6%)	101 (49.0%)	4.0 ± 1.29	High satisfaction
The instructor was supportive and responsive to my questions	20 (9.7%)	8 (3.9%)	14 (6.8%)	49 (23.8%)	115 (55.8%)	4.1 ± 1.29	High satisfaction
I was satisfied with the response time I received from my instructor	18 (8.7%)	8 (3.9%)	20 (9.7%)	52 (25.2%)	108 (52.4%)	4.1 ± 1.26	High satisfaction
I found it easy to participate in discussion questions	23 (11.2%)	9 (4.4%)	23 (11.2%)	58 (28.2%)	93 (45.1%)	3.9 ± 1.33	Intermediate satisfaction
I found it easy to communicate online with other students	18 (8.7%)	6 (2.9%)	23 (11.2%)	53 (25.7%)	106 (51.5%)	4.1 ± 1.25	High satisfaction
I would rate the overall value of the course as (excellent to poor)	0 (0.0%)	1 (0.5%)	20 (9.7%)	75 (36.4%)	110 (53.4%)	4.4 ± 0.70	High satisfaction

Mean satisfaction categories: (low 1–<3,intermediate 3–<4, and high 4–5.)

The high satisfaction levels indicate that students found the course well-organized, the grading system transparent, and the instructor support effective. The strong overall course rating suggests that the course was well received and met students’ expectations. However, slight variations in standard deviations suggest that some students may have had diverging experiences, warranting further investigation into specific areas for enhancement, such as more interactive activities or personalized feedback mechanisms.

Regarding the open-ended questions, 108 (**52.42%**) of the participating students answered them and described the course’s useful advantages and suggestions for course improvement. The thematic analysis of their answers resulted in three subthemes under each of them, as shown in Table 6. Under the course usefulness theme, the most recurring subtheme was *activity-related usefulness*, in which one of the participating students described the course activities as “We learnedhow to make an infographic, the importance of teamwork, and learning about many health and environmental matters” (student 47), and another student wrote, “Lectures on planetary medicine were valuable and interesting” (student 59). Usefulness related to educational content and knowledge was also expressed; for example, “I learned the importance of climate, its impact on the world and its risks” (student 67). Another student wrote, “I extend my sincere thanks to all the university professors. It was an interesting course that taught me many topics that I did not know and did not imagine were so important” (student 48). *Students’ growth and future action plans* appear in the description of the students, by saying, “knowledge gained shifting our focus to something that was usually ignored or put under the rug” (student 56). “It created more for me to discuss with the world” (student 51), and “It made me wonder more about health of the environment, and How can I help maintain it or repair it if possible?” (student 60).

**Table 6:** Distribution of themes and subthemes of students’ answers to open-ended questions(*n* = 108)

Theme	Subtheme	Description/example quotation	Cases *n* = 108	% cases
**Usefulness**	Activity related	Practical and interactive assignments and research activities	98	90.7%
	Educational content and knowledge	Course topics, research papers and data used as course materials and knowledge gained during the course	79	73.2%
	Action related	Students’ growth and future plans of using knowledge gained	64	59.3%
**Improvements**	Media related	Improvements related to using specific technologies or social media platforms suggestions	76	70.4%
	Educational content	Additional content and information related suggestions	58	53.7%
	Activity related	Improvements related to interactive and guidance about action-based course activities	42	38.9%

Under the improvements theme, *media-related improvements* were frequently mentioned by students. For example, a student suggested new media formats by saying “Lectures will be better if they are in another form than voice on ppt; mp4 videos andusing telegram better than WhatsApp” (student 7). *Content-related improvements* were explained by students who suggested adding additional topics and learning methods, as in the statement “Incorporating more practical application and real-world case studies” (student 87). *Activity-related improvements* included improvements related to interactive and action-based course activities, as evident by one student writing “maybe adding some instructions about how to start an awareness campaign about preventing climate issues” (student 82).

## Discussion

This study demonstrates that the online elective course on planetary health and sustainable healthcare significantly enhanced students’ knowledge and was well received, as evidenced by high satisfaction ratings.

### Knowledge Gaps in Planetary Health Education

Prior to the course, most students (93.2%) expressed limited knowledge about planetary health and sustainable healthcare and expressed the need for more information. Similarly, studies from Brazil and Germany found that many medical students were unfamiliar with planetary health but eager to learn more [3, 31]. A related study revealed that 79% of students had poor or terrible understanding of the environmental impact of clinical pathways before exposure to the topic [14].

In this study, social media (86.9%) and internet searches (81.1%) were students’ primary sources of information, with fewer students learning about planetary health from undergraduate courses (61.1%). A Brazilian study similarly found that 55% of students had learned about planetary health online, and 42% through formal classes [31].

Medical students widely acknowledge their responsibility in addressing climate change and promoting sustainable healthcare [14]. In this study, 87.3% of students agreed that medical schools should prepare them to tackle climate-related health challenges, aligning with findings from previous studies [3]. Additionally, 87.3% believed they could take active steps to combat climate change, and 92.7% expressed the need for proper training to fulfill their future roles as physicians. Similar studies report that students strongly support integrating sustainability into medical practice, with 97% agreeing that healthcare workers should help reduce environmental impacts [14]. The students’ written responses reinforced this perspective, with many expressing their future intentions to apply knowledge gained from the course to promote planetary health initiatives. Although this study assessed students’ sustainability practices in daily life, clinical sustainability behaviors were not explored, which remains an important area for future research.

### Effectiveness of the Online Course in Enhancing Knowledge

The course successfully addressed significant knowledge gaps, as demonstrated by statistically significant improvement of posttest scores over the pretest, as well as a majority of students reporting high satisfaction with the achievement of course goals and learning outcomes. Similar improvements have been observed in other online educational interventions. For example, an online planetary health module was piloted at Brighton and Sussex Medical School, targeting third- and fourth-year medical students. The self-directed e-learning course comprised four multimedia chapters covering core topics in sustainable healthcare, including climate change as a driver of healthcare sustainability, the environmental impacts of healthcare and carbon hotspots, principles of sustainable clinical practice, and the health co-benefits of climate mitigation [14]. Each chapter included interactive quizzes with answer explanations and links to additional resources. Of 316 students invited, 40 enrolled and 33 completed the module, resulting in an 83% completion rate, with 97% completing it within 90 min. Evaluation revealed that the proportion of students with good or excellent understanding of climate change and healthcare sustainability increased from 2–21% to 91–97% after completing this online module [14]. A comparable planetary health module was integrated into the compulsory “Basic-Clinical Integration 3” course during the third semester at a Brazilian medical school. The module addressed all five domains of the Planetary Health Educational Framework—Interconnection within Nature, Anthropocene and Health, Equity and Social Justice, Systems Thinking and Complexity, and Movement Building and Systems Change—and involved 96 students and 9 faculty members [31]. Key instructional activities included pre-recorded lectures, small group sessions, real patient interviews at the university hospital, and the development of portfolios that connected clinical cases to planetary health concepts across epidemiological, scientific, and health systems dimensions. Evaluation of the Brazilian intervention reported that 66.7% of students experienced significant learning, and 83% rated planetary health education as highly relevant to their training [31]. These findings reinforce that structured online modules can significantly improve students’ knowledge, attitudes, and confidence in addressing planetary health challenges.

### Impact on Attitudes and Satisfaction with the Course

Beyond knowledge acquisition, online sustainability modules have been shown to foster positive attitudes toward environmental responsibility. A compulsory sustainability in quality improvement workshop was integrated into the core third-year curriculum at Bristol Medical School, reaching approximately 342 students. The workshop combined sustainable healthcare principles with quality improvement methods, covering topics such as healthcare’s environmental impact, waste reduction, and quality improvement tools like process mapping and stakeholder analysis [32]. Activities included a central lecture broadcast and locally facilitated group sessions using case studies, pre-readings, and interactive discussions. Evaluating these workshops showed that 95% of students recognized sustainability’s importance for future healthcare, and 94.2% planned to reduce carbon emissions in their medical practice [32].

In the current study, students reported high satisfaction with the course structure, particularly appreciating the clarity of the grading criteria, the achievement of learning outcomes, and the instructor’s responsiveness and communication. The thematic analysis of students’ open-ended responses further highlighted three main areas of perceived usefulness. First, activity-related usefulness was noted, as interactive assignments were identified to enhance the learning experience. Second, the educational content and knowledge provided by the course were deemed essential. Third, students expressed a desire for action-oriented learning, indicating their intention to apply what they learned in real-world settings. Additionally, students suggested several improvements to the course, including enhancing multimedia content—such as replacing voice-over PowerPoint presentations with videos—expanding the curriculum to incorporate more practical applications and real-world case studies, and providing guidance on action-based learning activities like sustainability projects. In other comparable courses, students suggested improving interactive and engaging teaching methods and the use of multimedia resources and emphasized the need for real applications of healthcare sustainability [14].

### Addressing Barriers to Integrating Planetary Health into Medical Education

The online format of this course overcame common barriers to integrating planetary health into medical curricula, including limited faculty expertise and time constraints [6]. A study aimed to examine health professions educators’ knowledge, attitudes, self-efficacy, and teaching practices related to sustainable healthcare education across 13 health disciplines within a single Australian university [33]. In the Australian study, only 36.9% of faculty felt confident explaining planetary health principles, and 44.2% believed they could inspire students to incorporate these principles into their practice [33].

The online elective format of the course facilitated its integration into the busy undergraduate medical curriculum at Alexandria Faculty of Medicine as well as overcame the barrier of teaching large groups of students. Other reported barriers to the integration of planetary health into medical curricula are the lack of faculty expertise and limited time within the core curriculum [6]. A study in Australia reported that 36.9% of respondents felt confident in explaining planetary health principles, while 44.2% believed they could inspire students to integrate these principles into their practice [33]. Online modules provide a scalable solution, reducing faculty workload and allowing students to learn at their own pace. Additionally, open-access resources and online repositories can facilitate widespread adoption of planetary health education [6]. While the online format offered flexibility and scalability, it also presented limitations such as reduced real-time interaction, variable student engagement, and potential access issues. Asynchronous tools (e.g., recorded lectures, forums) improved accessibility but may not replicate the depth of interaction possible in face-to-face or blended settings. Future iterations could adopt a blended delivery model to enhance interactivity and peer collaboration.

Another limitation is the absence of a control group, which restricts causal inference and introduces potential threats to internal validity, such as history effects and maturation. To address this, a pretest–posttest design was used to evaluate within-group changes, with reliability supported by an internal consistency coefficient of 0.78. Future research should consider incorporating a control group to strengthen causal inferences and further validate the effectiveness of the intervention. The elective nature of the course resulted in a non-probability sample, which, while sufficiently large for analysis, represented only 8% of the third-year student population—raising concerns about potential volunteer bias. Students opting in may have had a prior interest in sustainability, possibly inflating engagement and attitudes. Future studies should aim for randomization or broader and diverse participation to enhance generalizability. Despite the findings not being generalizable, the detailed description of the course design, including its evidence-based pedagogical practices, enhances its transferability, allowing other institutions to adapt and implement similar educational interventions despite differences in curricular structures. Finally, evaluation scope is confined to the first two levels of Kirkpatrick’s model (reaction and learning) which provides a comprehensive valuable insight into immediate learning gains. Future research should consider implementing long-term evaluation studies to assess whether such interventions lead to behavioral changes in clinical practice, reinforcing sustainable healthcare principles.

## Conclusion

The online course effectively bridged knowledge gaps, received high student satisfaction, enhanced medical students’ attitudes, and reinforced students’ motivation to take actions toward improving sustainability in healthcare. The course adopted evidence-based online teaching methods, as the design was informed by the CoI model, and the evaluation followed Kirkpatrick’s framework, ensuring a structured and interactive learning experience. While the course is highly effective, there are several areas for potential enhancement. Enhancing multimedia delivery by incorporating videos and interactive simulations, providing more structured guidance on action-based sustainability projects, and expanding content to cover more real-world applications and clinical sustainability practices would help students engage more deeply with the content, solidify students’ learning, and improve their satisfaction. While the study demonstrated positive outcomes, it is important to acknowledge the primary limitation—the absence of a control group—which limits the ability to draw definitive causal conclusions.

### Implications for Medical Education at Alexandria University

The findings of this study have important practical implications for the Faculty of Medicine at Alexandria University, particularly as the institution continues to modernize its curriculum in response to contemporary global health challenges. The demonstrated effectiveness of the online planetary health module highlights a timely opportunity to formally integrate sustainability and climate change into the undergraduate medical curriculum, which currently lacks structured coverage of these urgent topics. While this integration could initially be achieved through an elective module, the long-term goal should be incorporated into the core curriculum to ensure broad student exposure at Alexandria University. Furthermore, it is recommended that medical schools incorporate planetary health education into undergraduate medical training to ensure future healthcare professionals are equipped to address environmental health challenges. Educational content should be tailored to institutional and local needs while aligning with global sustainability objectives.

## Data Availability

The datasets analyzed during the current study are available from the corresponding author upon reasonable request.

## Abbreviations

GMC: General Medical Council
ESH: Education for Sustainable Healthcare
CBL: Case-Based Learning
CoI: Community of Inquiry
COP: Conference of the Parties
UNFCCC: United Nations Framework Convention on Climate Change
MCQs: Multiple-Choice Questions
IREB: The Institutional Research Ethics Board
SPSS: Statistical Package for Social Sciences
CI: Confidence Interval
M: Mean
SD: Standard Deviation
QDA: Qualitative Data Analysis
MOH: Ministry of Health
